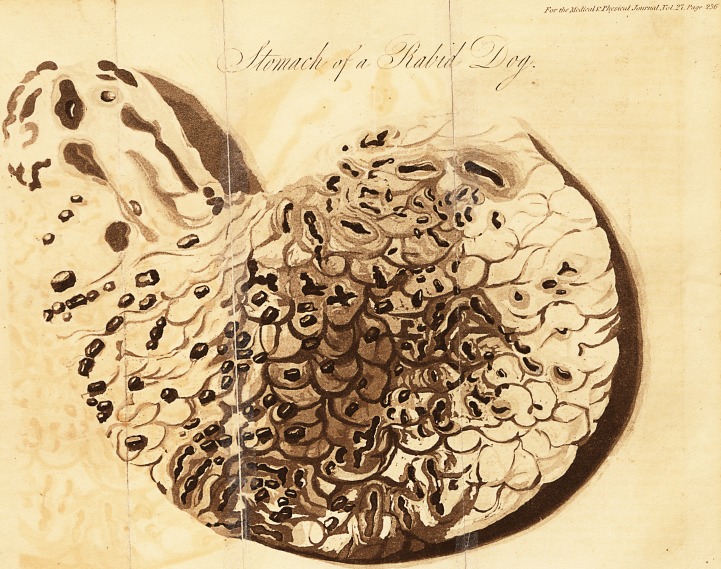# Book Reviews

**Published:** 1812-03

**Authors:** 


### CRITICAL ANALYSIS

### *OF RECENT PUBLICATIONS*

IN THE

### DIFFERENT BRANCHES OF PHYSIC, SURGERY, AND MEDICAL PHILOSOPHY.

*A Treatise on some Practical Points relating to the Diseases of the Eye.* By the late J. C. SAUNDERS, Founder and Surgeon of the London Infirmary for curing Diseases of the Eye. *To which is added, a short Account of the Author's Life, and his Method of curing the Congenital Cataract.* By

By his Friend and Colleague, J. R. Farre, M.D. The whole illustrated by colored Engravings. 8vo. pp. 216. Longman and Co. 1811.

THE danger of anticipation by fair and lawful means, where an author has useful matter to convey upon a topic of extensive interest, is not the real evil of a protracted publication. Neither is the common but malevolent insinuation, that the communication is retarded from interested motives, a circumstance ultimately injurious to the character of the writer or of his production. Though the interval between the announcement and the appearance of his work should extend even to the ninth year, provided that interval be employed diligently and faithfully in weighing and revising opinions, which when known will be acted upon to the infinite benefit or prejudice of a vast portion of the community, we are prepared to assert the wisdom and benevolence of his determination. But there is an inconvenience attending delay upon a topic of great and general interest, which it requires a little more discernment to notice, and a little more candor to overcome, than the public voluntarily exert in behalf of an individual. This is a delusion that men commonly practise upon themselves after busying their minds upon given subjects, which insensibly persuades them that they have been long familiar with facts and opinions which they have very recently taken up and adopted. Few books relating to the medical profession have created more prospective interest than the present. This has not been confined to the profession, for the obvious reasons that the benefits of the author's experience, already widely diffused, were unequivocal and demonstrable to all classes and capacities; that he had himself founded the institution from which he derived his means of usefulness; and that by his great personal merits, and conciliatory attentions, he had secured the opinion and confidence of the poor, and the support and approbation of the opulent. These were circumstances to raise public curiosity, and especially that of the profession. Since his institution was founded, his modes of practice have been inquired into with a steadily increasing eagerness, and of late have been canvassed with much freedom. Some who had witnessed his operations, raised upon the imitation of them, and we believe upon this alone, popularity and emolument in the provinces; and since the commencement of the year 1811, the practice of this dispensary has been public, and attended by many students of surgery. Thus the leading features of the work have become familiar before its publication, and we are told that this this point is nothing new, that the other is old; that Mr. A. and Mr. B. have long performed that operation precisely in the same way: nay, those very gentlemen have at length boldly stepped forward to assert it. We pay no regard to these after-statements, and utterly disclaim the spirit in which they are conceived. The only admissible evidence is the written record of fact; and we doubt if, trying the author by this standard, it can be shewn that he has been of the class of servile imitators or subtle plagiarists; if any difference shall remain in the minds of honest readers and competent judges on the justness of the author's claim o a reputation which will outlive all attempts to slander it.

First Chapter.—"*On the Inflammation of the Conjunctiva in Infants.*"

This name conveys Mr. Saunders' view of the disease, hitherto known by the name of 'the Purulent Ophthalmy.' Scarpa has very lightly touched on this subject, and the only respected authority among the surgeons of this country is that of Mr. Ware. This gentleman appears to have described and treated one of the symptoms of the disease, as if constituting it. He seems to have overlooked the relation, between the inflammation and the discharge, of cause and effect. He distinctly states the first stage of the disease to be "an increased discharge from the minute pores of the conjunctiva;" and attributes the subsequent affection of the cornea to the eroding quality of retained matter, joined to "the pressure of the swollen eye-lids." He speaks of "the cornea having been known to burst." The indication of cure, agreeable to this hypothesis, consists in "immediately constringing the relaxed vessels" by strong styptic injections. These are designed to "thin the conjunctiva," and "check the redundant discharge." Such is Mr. Ware's pathology of the Purulent Ophthalmy, in his tract on that subject republished in 1805.

We cannot but regard Mr. Saunders' view of the matter as greatly more consistent with the intelligible and acknowledged principles of pathology. He states the disease to consist in an inflammation of the conjunctiva, which is affected much in the same way as the membrane of the urethra in gonorrhœa. He therefore advises that the strict antiphlogistic plan should precede the use of injections, and that the injections, when the activity of the inflammation has subsided, should be mild astringents. He presents us with a scientific, and as far as our limited experience qualifies us to judge, a very accurate, picture of the progress and termination of the disease. In its vehement form it tends to sloughing sloughing either of the whole cornea or of portions of it successively: the ulcer left by the slough may in time become sloughy, or it may extend itself by the ulcerative process: in the former case, tonics, or the extract of bark, in pills, may be given freely with advantage; in the latter, the case resolves itself into that of ulcer of the cornea, and will require the same local treatment as the author recommends under that head.

In the treatment of the inflammatory stage, the use of leeches is preferred to the practice of scarification. A grain of calomel, and a little rhubarb and magnesia, are required occasionally to correct the disordered state of the bowels. The opacity which threatens slough is described in terms of distinction from that which indicates the process of healing, and the excision of the morbid portion of conjunctiva is recommended for the cure of that obstinate aversion of the eye-lids which sometimes follows the disease. Mr. Saunders confutes the notion that an acrid quality of the matter retained between the lids, produces the effects attributed to it; indeed, he proves that it possesses no such property. In one case the discharge was retained for several days within the closed lids, by compress and plaister, so that the eye was immersed in the discharge; in which time three ulcers of the cornea healed, and the discharge ceased: besides, the discharge is often most copious where no breach of surface ensues.

There is but one point in this essay upon which, after a careful perusal, we are disposed to differ a little with the writer. He considers the inflammation to be of the erysipelatous kind, from its coincidence in rapidity, extent, and the production, of sloughs. With the fact of its production of slough, we are perfectly satisfied, and believe that the eye never suppurates from this cause, as the vulgar notion has been. But we question if this point is sufficient to establish the identity. We admit that morbid appearances are modified by differences of texture, and therefore do not build an objection upon the difference between those of the erysipelas of the cutis and the conjunctiva. But why may not the purulent be a simple inflammation of the conjunctiva, of which the destructiveness is proportioned to the violence? We know that actions which do not differ in kind differ in degree; and that they produce, under such circumstances, a corresponding difference of result. Thus inflammation of the peritoneum terminates in lymph, and more rarely in pus; but, when most vehement, in gangrene. Of the mucous lining the larynx, alimentary canal, and bladder, the milder form of inflammation produces pus; the violent, in the former case, lymph; in the two latter, gangrene.

We

We should say then, that the mild and manageable inflammation of the conjunctiva, of which the purulent discharge is a symptom, was that which terminated in deposition of lymph (opacity) with or without superficial ulceration; the vehement and less manageable form, that which produced sloughing of the cornea. In symptomatic character, the former may be compared to the mild, the latter to the virulent, gonorrhœa; and in treatment the same analogy must hold. The surgeon, for example, who applies (according to our conception of the disease) a stimulant injection in the active stage of the ophthalmia, should, to be consistent, employ the same in a clap, where the lips of the urethra are angry and tumid, and the scalding excruciating. This execrable practice has had its day.

Second Chapter.—" *On Inflammation of the Iris.*"

This is unquestionably the best account of the Ophthalmia Iridis which has yet appeared; indeed, it is the first full and distinct delineation of this formidable disease. The description cannot be abridged with justice to the author, and is too copious to extract. Considering how strongly the disease is marked, and that the conjunctiva frequently remains unaffected, and when participating, is only affected secondarily or by sympathy, we are surprised that it should not sooner have received a separate and circumstantial consideration. It terminates, if left to take its course, in obliteration of the pupil, already greatly contracted, by coagulable lymph. It often happens that the capsule of the crystalline lens partakes of the inflammation, and uniformly the margin of the pupil becomes fixed by adhesion to the capsule, whether the latter turns opaque or not. We must not trifle away time in such cases. Leeches and laxatives, and lotions and regimen, are confessedly inadequate to reduce the strong adhesive inflammation. Blood must be drawn in quantity from the system; whether from the forehead or the arm, Mr. Saunders thinks is of little moment, and we think so too: enfeebling medicines, as tartar emetic, must be given to cooperate with the lancet, if the system be such as to require it. When the danger of disorganisation by the violence of the inflammation is overcome by these means, our attention is drawn to the contracted and motionless pupil. The power of the narcotic class of vegetables, to dilate the pupil when free to move, is now well known. That of the Atropa Belladonna is most conspicuous.

In England Mr. Saunders was, we believe, the first who employed this substance in the treatment of ocular diseases. He conceived with admirable ingenuity the idea of dilating the the pupil by the belladonna in the recent ductile state of the lymph, which had been effused from the inflamed vessels of the iris upon the crystalline capsule. He has communicated in this paper several examples of his success in executing this happy project. Estimating highly, as we feel inclined to do, the value of this discovery, it is matter of grateful reflection to us that it first met the public eye in the pages of the Medical and Physical Journal, in 1806.

We regard this paper as a *chef d'œuvre* of its kind. The importance of the thing to be done, contrasted with the simplicity of the agent, the ingenuity of the proposal with the facility of its accomplishment, gives it an air of perfection which cannot fail to strike the mind of an intelligent reader.

The belladonna, regarded in the full scope of its application to practice, is in our opinion an instrument of inestimable value. More than a hundred years ago its action upon the iris was announced in Ray's Herbal, but the hint as well as the herbal was forgotten. Such is the progress of knowledge. The discovery of a fact and its application are often separated by a much longer interval: a reflection which should teach us not to hold lightly the results of observation, of which the use is not obvious. Mr. Saunders embraces the general opinion of a double set of fibres in the iris, and supposes the belladonna to act upon the radiated order. We are distrustful of the anatomical fact, and still more so of the physiological explanation. But, as the argument, although not devoid of interest, is at present speculative, we shall not enter upon it here.

Third Chapter.—"*On the Cure of the Inversion of the Upper Eyelid, by Excision of the Tarsus.*"

The distressing and disfiguring disease which results from an inversion of the eyelid is described in this essay, after some general remarks on the structure and uses of the part. In the earlier periods of the disease the operation of Dr. Crampton is approved; but, in the ultimate and inveterate state, Mr. Saunders performs the extirpation of the whole cartilage, leaving only the puncta lacrymalia. This, he assures us, he has executed with the most happy results. From insertion of the levator palpebræ muscle into the integument and conjunctiva, which important fact was pointed out by Dr. Crampton, the patient retains the power of elevating the lid to a height sufficient to clear the pupil; and the deformity is slight, compared with that occasioned by the disease. The operation consists in stretching the eyelid upon a convex horn or silver plate, and then dissecting the cartilage out through an incision in the skin and orbicularis palpebrarum, immediately behind the roots of the cilia, and extending from the puncture to the external angle. The wound requires no dressing.

In partial inversions of the tarsus which are not relieved by the extraction of the cilia, a piece of skin containing the roots of the cilia may be dissected out. The inversion of the lower lid arises from various causes: from ulceration of the tarsus altering its shape from encysted tumors between the conjunctiva and tarsus, from the morbid enlargement and protrusion of the conjunctiva connecting the lid with the globe, in which case it forms a roller for the tarsus to turn upon, and lodge against the eye. If our endeavors to keep the lid in its place, with the reduction of the inflammation, is insufficient for the cure, Mr. Saunders advises the excision of this fold of conjunctiva, and a compress to carry the orbital edge of the tarsus inwards. The closed and open state of the lids after the excision of the tarsus is represented in the plates, and confirms the statement of the author.

We concur in opinion with Mr. S. that the disease is most formidable when arrived at the pitch which renders such an operation expedient; but we doubt if either this or any other proposal for its cure is followed by that distinct and lasting benefit which should entitle us to speak sanguinely of its merits.

The remaining chapters are compiled from the same sources of observation, by the editor, with the frequent introduction of the author's MSS. notes and cases.

Fourth Chapter.—" *On some of the more important Terminations of Ophthalmia.*"

1. By effusion of coagulable lymph.—This may take place between the conjunctiva and cornea; between the lamellæ of the cornea; between the cornea and iris; or even between the iris and capsule of the crystalline lens. At uncertain periods the lymph becomes organized. The adhesive inflammation is described in the Essay on Inflammation of the Iris, and particularly the changes induced upon the pupil. The diagnosis between the simple and the syphilitic ophthalmia iridis, which greatly resemble each other, is here laid down, and some excellent cases are added to illustrate the efficacy of mercury in the latter disease, and consequently to prove the importance of the distinction. The simple inflammation of the iris yields to active depletion, the specific does not; and, if mercury is not timely employed, terminates in the destruction of the organ. The

 appearance appearance of the eye in the curable and incurable states of syphilitic inflammation is represented in the plates.

2. By suppuration.—Sometimes a little abscess forms in a patch of lymph deposited by inflammation on the cornea. If it opens internally, pus is seen in the anterior chamber. It requires a nice observation to distinguish soft lymph and pus, the best criterion is the figure which the effused matter assumes; "the lymph rises in irregular masses, the pus maintains a level."

3. By slough.—This section is in a great measure anticipated by the chapter on inflammation of the conjunctiva. The identity of the purulent ophthalmy, in the infant and adult, is established by cases illustrating the common termination by slough of the cornea. This section contains further remarks, denoting a habit of minute and patient observation, on the appearances characteristic of the sloughing process; and concludes with cases to shew the efficacy of the cinchona in checking it, and promoting the adhesive inflammation.

4. By ulceration.—Pustules of the conjunctiva, resembling aphthæ, are a common form of strumous ophthalmia; and, when appearing upon any part of its surface, terminate in ulcers of the cornea. When the ulcers are indisposed to heal, a solution of the nitrate of silver (Scarpa uses it in the solid form) should be injected upon them in a stream from a syringe. The quantity of lymph deposited around the ulcer may be more than the healing process requires; this must be corrected by the antiphlogistic treatment. Some very illustrative cases follow to explain the circumstances in which the tonic plan, medical and topical, is indicated, and the period at which it should be taken up. This chapter is of peculiar value, as it unfolds in a systematic view, and upon scientific principles, the results of inflammation attacking this delicate organ.

Professor Scarpa's descriptions are those of a well-informed and practical surgeon, but the connection of these subjects, under the general head of inflammation, is more simple, and clear, and impressive, than distinct delineations of nebula, hypopion, ulcer, &c. as distinct from ophthalmia, and from each other.

Fifth Chapter.—"*Illustrations of some of the more important Changes of the Structure in the Eye.*"

This paper contains some very valuable histories of amaurosis, followed by tumors, and other marks of disorganisation consequent upon the morbid state of the retina, or by fungus of a malignant character, which unhappily do

 not not stop at the destruction of vision. The morbid appearances accompanying three several cases of this terrible disease are represented in the plates, and the account of the dissections is given in the words of Mr. Astley Cooper. From these it would appear that the optic nerve and retina, are not the parts originally diseased. In two of them the disease appears to have been seated between the sclerotica and choroid, and in one by ulceration of the former to have made its way out among the contents of the orbit.

In the third dissection the vitreous and crystalline humors formed the disease, and were still invested by the retina. In the case formerly communicated by Mr. S. to Mr. Wardrop, and here reprinted, which case it should be observed refers to the fellow eye of that dissected and last described by Mr. Cooper, "the disease had extended in the course of the optic nerve to the ganglion, the whole of which was converted into a bloody tumor, too soft to be analysed by the knife, and which melted, as it were, under the touch, although the examination was made shortly after death." The editor, with a temperance strikingly characteristic of mature judgment, has refrained from offering an opinion on the origin and character of these morbid growths, and has not even ventured to distinguish them by any other than the general appellation of malignant fungi. We could wish that the modern zealous inquirers into morbid structure, would be on their guard against the premature use of names. It impedes instead of quickening the process of classification, which must always and of necessity be a work of time. Further histories with dissections will give additional value to these interesting documents, but it is a melancholy task to employ the mind upon; for it appears that even the earliest extirpation of the diseased organ is too late. The disease re-appears in other parts of the body, the viscera probably become affected, and the patient lingers and dies. The examination of the remaining chapter "on the congenital cataract," we reserve for our next number.

---

*A Dissertation on the Bite of a Rabid Animal; being the Substance of an Essay which received a Prize from the Royal College of Surgeons in London, in the Year* 1811; *by* JAMES GILLMAN, F.L.S. Member of the Royal College of Surgeons in London. 8vo. pp. 181. Callow. London. 1812.

THE universal fatality, the obscurity, if not the uncertainty, of the pathognomonic signs, especially as they arise in the dog; the discrepancies of opinion respecting the

*modus*

*modus operandi* of the poisonous or infecting material; and the little light that examinations *post mortem* have afforded, combine to render Rabies Canina a subject of great and important interest to the medical inquirer. As a question for philosophical discussion, as an illustration of the laws of life, and of the properties and principles of the mysterious power which impels and directs the movements and functions of organised beings, this disease also presents incitements of high consideration. Such inquiries have a legitimate claim, not only on philosophy and science, but on all branches and orders of society. Every disease over which medicine has failed to manifest a remedial power, has appropriate to it, the cunning assert and the credulous believe, some never-failing nostrum: *Rabies Canina* has its thousand specifics. Memory sickens at the destruction which has followed the employment of these *specifics.* And it is on this point that we view Mr. Gillman's Dissertation as a public good. It establishes the fact that no remedy has been yet discovered for the disease produced by the rabid poison acting on the system; and most forcibly shews that the only means which can be employed, with any chance for success, are those which remove the poisonous fluid from the part to which it is applied.

In treating this important subject, the author of this Dissertation distributes his materials into three sections, in which he examines the "Characteristics of Rabies in a Dog;" the "Treatment of the Bite of a Rabid Animal;" and the "Consequences of that Bite."

In the first section we are presented with many curious and interesting details on the distinguishing marks, constituting the character of rabies in the dog; a subject hitherto not sufficiently investigated, embarrassed with erroneous statements, false opinions, and hasty conclusions. That multitudes of dogs have been denounced as laboring under this disease when it did not exist, and prematurely destroyed in consequence, no doubt can remain. That serious, though false, alarms have arisen from this mistake, is equally certain; and that persons bitten by dogs thus circumstanced, or having only had their saliva applied to the unabraded cuticle, have severely suffered from nervous affections induced by disturbed imagination, and imitating closely the specific disease, cannot, we think, be denied. A more important fact even than this, however, is connected with our ignorance of the pathognomonic signs of rabies as it appears in the dog. We shall find, as we follow Mr. Gillman in his inquiry, that one symptom which has generally and popularly popularly been considered as marking this disease, does not invariably or commonly exist; and that a dog is actually suffering from rabies without being suspected, from the absence of a symptom which has given one synonym to the complaint.

To understand the history of this dreadful malady with a precision that may be practically useful, it is essential to trace it from its source downward, and to begin the investigation with the genus of animals where it originates. Though rabies has appeared in one individual of another genus of the class *mammalia*, it seems consonant with propriety to confine the investigation to the genus *canis;* and to shew distinctly the progress of symptoms in the dog. And, that we may not deviate from the graphic quality of a picture evidently drawn from the observation of nature, we shall give the detail in the words of the author, who, though he acknowledges the difficulty of giving a correct idea of the first symptoms that take place in this formidable malady, states the following phenomena, descriptive, as far as they go, of the progress of a case of rabies in the dog, to have fallen under his own notice.

"The animal always shows some marked deviation from his accustomed habits; a symptom which ought to be particularly regarded, and is most frequently not only a leading feature, but often an infallible proof, of approaching rabies. In the more domesticated animals, as lap-dogs, some strange peculiarities have been observed; as the picking up of the different little objects, such as paper, thread, straw, &c. or any thing which may happen to be presented to their notice. They have sometimes been observed to eat their own excrements and lap their own urine: these last, perhaps, are the strongest proofs of rabies, and should place us very much upon our guard, as this depraved appetite seems peculiarly to mark this complaint. Still, however, in this stage of the disease, they seldom attack any one unless provoked to it.

"It must be observed, that though a dog's temper remains meek, and frequently continues so during the whole of the disease, yet he is easily alarmed. He often observes the same obedience to his master, and shows the same degree of attachment, but still he is extremely irritable, and always treacherous, suffering any one to fondle him, and then suddenly snaps or bites with almost the least apparent provocation. As the disease advances, his eyes sometimes become inflamed, and a purulent discharge issues from the lids. The *pharynx* in some cases has been known to become so much inflamed as to render him incapable of barking. This symptom by sportsmen has been particularly regarded, and by them termed dumb madness. When deprived of this power, he makes a dismal howl, which is so well known, that when once heard it cannot be mistaken; nevertheless this is not an universal symptom. The incipient stage of of this disease has been marked by many writers with the loss of appetite, indifference, listlessness, and melancholy, which have been considered as strongly indicative of rabies; but they are symptoms that cannot be relied on, and attend many other diseases to which dogs are liable. Neither have they the least dread of fluids, and frequently eat with a voracious appetite.* I have had several patients under my care who were bitten by dogs decidedly rabid, and which both ate and drank a few minutes before and after they had committed the act;—consequently such statements should not be relied on.

"As the disease advances, the animal becomes extremely anxious and impatient, and has an inordinate desire to gnaw every thing around him. He is now seized with a more than usual antipathy to cats, which he bites if within his reach. When chained or confined, he makes the greatest efforts to break loose; and, if he succeeds, he wanders about seeking other animals to bite, but more particularly some of his own species. From a bite in this particular stage of the disease, the consequences are most to be dreaded, and the greatest care should be taken to avoid him. It has been a generally received opinion, that he never moves out of his road to bite any one: but this apparent indifference never takes place till he is exhausted by the disease, or rather till he becomes incapable of the effort; for even now, while he is most active, he is seeking industriously different objects to bite, to which his attention appears solely directed. It has been before remarked that he does not avoid water, and frequently laps it greedily: still in this stage of the disease he is often without the power of swallowing it. Another and not an unfrequent attendant of rabies, is inflammation of the bowels, which may be considered as having taken place when the animal is observed sitting on his rump in apparently great pain; very often he has the appearance of being paralytic behind.

"In the last stage of this disease all the preceding symptoms are aggravated: he now becomes extremely feeble; his jaw drops as if paralysed, and the saliva runs from his mouth; he wanders or rather

---

* This is a fact of much importance, as it points out the dangerous policy of considering *hydrophobia* as a leading characteristic symptom of rabies. Mr. Gillman's observation is corroborated by other practitioners. Dr. Clarke, of Nottingham, relates a case, which occurred in that neighbourhood, of a dog that was not suspected to labor under rabies until ten days after he had bitten an unfortunate person, *who in six weeks after the bite died of hydrophobia.* This dog *ate* and *drank* heartily, showed no signs of indisposition, hunted as usual, and occasionally went into a neighbour's house among children without injuring any of them; but on the morning of the tenth day (that is ten days after communicating the disease by the bite, and when he had no hydrophobia) he was observed snapping at every dog in the street, and was in consequence destroyed. Vide also Hunter's case of Master Rowley.

staggers staggers about with scarcely the power of biting; and, exhausted by the disease, dies generally on the fourth or fifth day from its commencement.

We have here a relation of the progress of this malady, comprehending many striking circumstances, as they were presented to our author; but we doubt if observation of the symptoms as they arise, of their degree of intensity in the different stages of the complaint's progress, of their giving way to or becoming mixed with others as the disease advances, has been applied with sufficient correctness; or if experience has yet been enough extended and matured to furnish materials for a history of *Rabies*, discriminated and characteristic; distinguishing it from all other diseases, and especially some others of the class *Neuroses*, so delusively imitative, as frequently, we are persuaded, to have been mistaken for, and then furnishing cured cases of the (supposed) specific disease.* That this is yet the state of our knowledge, Mr. Gillman admits, when he remarks that the "symptoms which accompany this disorder have such variable aspects, and are so diversified, that he is afraid, upon attentive examination, there are few which can be considered essential, or which belong to it exclusively; and the greater part are, perhaps, secondary symptoms only, such as are common to other diseases, or casual and of uncertain occurrence; some arising out of previous symptoms, others the effect of adventitious circumstances."

Are we then to understand that Nature has neglected to place her essential stamp, her unobliterable hand-writing on this malady? Not so:—the physician is not yet enough learned in her phenomena, and covers his want of discernment with the opinion that she is here irregular, uncertain, adventitious, and indiscriminating. Let us pursue the path in which Mr. Gillman has ably started; apply our investigation to the original source—the *dog*, and, perhaps, the desired development is not far distant. That we may aid the *dénouement*, we shall present to our readers a specimen of

* While hydrophobia shall be admitted as the pathognomonic mark of rabies, this mistake will happen. We could refer to numerous cases of spontaneous hydrophobia in confirmation of this, but we confine our reference, at present, to one printed in this Journal (vol. xxii. page 113), strongly marked, and terminating fatally on the third day. In this instance, all the symptoms had such a degree of intensity, the hydrophobia was so unequivocal, and the fatal termination so speedy, that without the most positive evidence to the contrary, it would become an established case of genuine rabies.

the the investigation we mean, as shewn in the following clearly written case, containing the history and progress of rabies in a dog, verified by the effect of his bite upon other animals; and the dissection of the dog, with that of the creatures his bite had destroyed.

"June 22d, 1811. A yard-dog belonging to a gentleman in Highgate killed yesterday one of his fowls, which he carried into his kennel. His master, when he saw it, put his hand into the kennel, took it out, and at the same time beat him: he made no attempt to bite him. The dog was not observed to be out of health, and, as was the usual practice at night, was unchained, and suffered to run about the stable yard, in which his kennel stood: on the following morning he was found in the pig-sty worrying an old sow and her two store pigs about ten weeks old, which he had bitten much, particularly about the ears. A suspicion now arose that the animal was rabid, and was ordered by his master to be shot. Being informed of the circumstance by a friend, I immediately went to see him. I found the dog without the least inclination to be violent or bite; on the contrary, he was shy, and appeared to be apprehensive of a second beating. This passiveness was observed, however, not to be his natural character; for when in health, if strangers entered the yard, he barked and was extremely violent: this variation from his usual habit placed me on my guard. There was, however, evidently much debility about him: he was thin, and had from one eye a slight purulent discharge; he lapped milk freely, and took animal food. I requested, however, to see the termination of the disease, which was granted; and therefore it was allowed to take its course. In the evening he took his food as usual; and it was remarked by the person who fed him that he had not differed since the morning, nor could we perceive that he was ill. The next morning he was more enfeebled, and began to refuse his food; in every other respect he appeared as yesterday: the third morning he was still more enfeebled, and paralytic in his hind legs; he also refused his food. He attempted once during the day to walk the length of his chain, which he could scarcely accomplish; and with the utmost difficulty he crawled back to his kennel. In this state of extreme debility and passiveness he laid the next day. On the fourth morning the gardener saw him so early as four in the morning, at which time he could scarcely discern his breathing; at breakfast-time he found him dead.

"The following were the appearances on dissection. The pia mater was slightly inflamed; the under surface of the epiglottis was also inflamed; the trachea and œsophagus exhibited no morbid appearances; the stomach contained a chocolate-colored gelatinous-like fluid; the villous coat was very generally inflamed, and several of the rugæ were livid, and of a chocolate color; there were a great number of mortified spots, some having the appearance of flattened black currants; some more raised like pustules; and in some parts the villous coat was ulcerated and destroyed. No other parts exhibited any morbid appearance. The stomach of this animal presented such determined marks of disease, that I have subjoined a plate* to endeavor to illustrate these appearances. As I wished to prove, if possible, whether the fluid contained in these apparent pustules had the power of infecting other animals, I inoculated two rabbits with it in several places, but without producing the disease or affecting the health of the animal, apparently, in the slightest degree.

"One of the young pigs, already referred to, which had been bitten least, and that only about the ears, on the morning of the tenth day after the bite, refused his food. When offered to him as usual, by placing it in his trough, after smelling at it, he ran back, pointed his nose in the air, and was much agitated: in the evening he had a convulsive motion, and twitching of his limbs. The eleventh day, (the second of the attack) he became extremely violent: when I saw him there was a considerable quantity of frothy saliva about his mouth: he started, and threw himself about in an extraordinary manner; sometimes he sprang at least three feet from the ground; then beat himself forcibly against the wall; and sometimes ran round on his hind legs, as dogs do when playing with their tails. This he continued till exhausted, he would fall down and pant; but soon again became convulsed, and leaped from the ground as before, falling with considerable violence on his back or sides. About noon, the person who fed him gave him a slight blow on the head, and killed him. On dissection the only parts inflamed were, the under surface of the epiglottis, and the villous coat of the anterior surface of the stomach to the extent of the palm of the hand. The head had suffered such injury from the violence of the convulsions, that no dissection could be made: the skull and lower jaw were fractured: tongue in its natural state.

"The other pig was seized on the noon of the fourteenth day after the bite. This animal was considerably torn by the teeth of the dog, and had a deep wound on the back between the shoulders. He was first seized with rigors, and stood shivering beside his trough, rubbing the bitten parts, which I had observed him to do also the day previous. He refused his food, and appeared debilitated, moving himself languidly and feebly. On the second day of his illness, he became paralysed in his hinder legs; and, after crawling, which he did in the morning, from his sty, in a few hours he

* The liberality of Mr. Gillman has permitted us to copy this plate. We are not aware, that the appearances exhibited in it have been described and presented to the public before the printing of his Dissertation. We are informed, however, that, at the latter end of January last, a man died of rabies, whose stomach presented similar appearances. We forbear to go into a detail, because we would not anticipate the gentleman who watched the progress of the disease, and examined the body after death, and who will, we trust, publish an account of it.

was was so much worse as to be unable to return. He lay on his side frothing at his mouth, rubbing his nose on the ground, and pulling the straw about and breaking it with his fore-feet and teeth the whole day; he made frequent attempts to swallow some of the bits of straw, in which he very seldom succeeded. The eye-lids were much separated, which gave a staring appearance; the conjunctive membrane was much inflamed. When the old sow went near, it made the same familiar noise or grunt: it did not appear to have the least inclination to bite any thing; I tried it repeatedly by putting a piece of stick into its mouth. The pupils of its eyes were, I thought, dilated, but it could see very well, and was alarmed at the motion of a stick when within two yards of it. On the third day, the seventeenth from the bite, it lay the whole time on its side; and, except occasionally slight twitchings in the legs, it remained perfectly still and unable to rise, having lost all power in its extremities; it squeaked when touched, as if the skin was more than usually sensible, and particularly when the mother went near it or touched it. Towards the evening the breathing became so feeble as scarcely to be discerned, and the conjunctive membrane so turgid as to protrude beyond the palpebræ. It died late at night seventeen days from the bite.

" *Appearances on dissection.*—On examining the brain there was considerable effusion of blood from the veins of the pia mater, contiguous to the superior longitudinal sinus; there was also much inflammation of the pia mater itself; no morbid appearances to be seen on the tongue; the under surface of the epiglottis was inflamed, as in the preceding case; in the œsophagus there was no disease; the inner membrane of the posterior surface of the trachea was very slightly inflamed; the villous coat of the anterior surface of the stomach towards the cardia was also very slightly inflamed; and the surfaces of two of the rugæ on the opposite side of the stomach presented a livid appearance, one more inclining to a chocolate color, approaching to mortification; the bladder was very much distended with urine, containing more than a pint. The tunica conjunctiva was turgid, and protruded beyond the palpebræ.

" From this last pig two rabbits were inoculated with the saliva, but without producing any effect. Twenty-seven days after the bite I observed the old sow taking up in her mouth the dirty straw and filth that lay about the sty, which immediately gave rise to a suspicion that this peculiar disease was approaching. The following morning (twenty-eighth day) she refused her food, was perfectly quiet and harmless, came from her bed when called, and was seen to rub very often in the day the wounded parts upon her ears. In the evening having some ripe gooseberries in my hand, I went into the sty, (as I was willing to tempt her to eat, for since the morning she had not taken any food,) and offered her them: she ate a few, about half a dozen, and picked up by my feet several skins or husks which I had thrown down; she had, however, much difficulty in swallowing these small bodies.

" The next morning (third day of the attack) she appeared nearly the same as the preceding evening; she was dull, but had no paralysis in her extremities, and came out of the sty when called. There was some inflammation about the parts which had been wounded by the dog's teeth, and more so about those on the ears. I went again into the sty and offered her some milk; she made several attempts to drink, but could not; there was a peculiar convulsive motion of the head and twitching of the under jaw, but no dread of fluids, as she took up with her teeth, apparently observing much caution, some small pieces of cabbage-leaf which swam on the top of the wash; but after moving them two or three times between her teeth she suddenly dropped them, unable to chew or swallow them; she walked steadily, but moved herself very slowly; the eyes had the natural appearance. By the evening this disorder had made much progress: the convulsive twitchings of the head were much increased, and she was extremely restless; for when this peculiar motion of the head from side to side had ceased, she was busily employed in grubbing up the earth with her nose, as if in search of food. When the spasmodic motion returned, (which it did once in a quarter of an hour or twenty minutes,) she squalled out and became alarmed when any one approached her. On the fourth morning all the preceding symptoms were increased, and every hour the paroxysms returned oftener and were more violent. She frequently jumped up suddenly on her hind legs, and threw herself upon her back with considerable violence: she was affected by the least noise; when I stamped with my foot firmly only the ground, she was thrown by the noise into the most violent convulsive state, and squalled horribly. To such a high degree, in short, did the morbid excitability of the nervous system arrive, that the poor animal was affected by the least touch, which seemed to be torture. In the evening the symptoms were still more aggravated: she beat herself against the walls, and sprang up against the roof of the sty. These attacks continued, and were repeated about once in every ten minutes, till about two o'clock in the morning, when I ceased to hear them, and when I arose I found her dead.

"*Appearances on dissection.*—Some slight marks of inflammation about the epiglottis: the villous coat of the stomach was inflamed toward the pylorus, and had several chocolate-colored striated marks approaching to mortification."

This account, drawn faithfully, as it appears, from nature, gives the history of rabies in some of its most interesting points. On the sometimes-controverted fact of rabies arising spontaneously, it seems decisive. A dog, chained in a yard, without intercourse with animals capable of inoculating upon him this disease, has it in its genuine form, verified by the effect produced by his saliva. This animal seems never to have been affected with the intense spasms and irritability commonly accompanying rabies; nor with a disposition to do mischief, except on the second night, when he worried the pigs: on the contrary, under the influence of the disease, he became more passive than was natural to him. He was never hydrophobic, but died on the fourth morning from the attack; and on examination of the body, the stomach was found to exhibit morbid changes, greater than had before been seen to result from the action of this poison. The bitten pigs, although belonging to a genus in which rabies is not known spontaneously to arise, were much more violently affected, particularly the sow, with spasm and convulsions, but they had not hydrophobia. In them the disease rapidly reached its termination; but the marked character seen in the stomach of the dog was wanting. From these cases we should be led to the conclusion, that sometimes death is occasioned in rabies by visible mischief done to some vital organ; at other times by an affection of the brain and nerves, exhibiting on dissection no organic lesion or apparent morbid alteration. In one class the symptoms will be langour, debility, passiveness, paralysis, death; the dissection will shew inflammation and sphacelus. In the other, languor, spasm, irritability, intense morbid sensibility, death: if the dissection shews appearance of disease, it is of a minor kind, and esteemed inadequate to the production of such symptoms, or of occasioning death. We are ready to allow that this generalization may be premature, and we venture it only as a hint. But we can understand that gangrene in the stomach may occasion death, and anticipate the frightful spasms we have seen occur in rabies; and we can without difficulty comprehend that when death results from convulsions, and has not the intervention of mortification, that every symptom will be more vehement. This, however, we must not consider as indicating any particular modification in the essence of the disease: it may depend on adventitious circumstances separate, or combined with idiosyncracy. The great advantage of remarking these facts is, that they prove rabies to exist independent of hydrophobia and convulsion. We have before shewn, by a case cited from our Journal,* that hydrophobia, in its most violent form, does also arise independent of the rabid poison. To correct the false idea of hydrophobia being an essential or pathognomonic symptom of rabies, we have dwelled with some solicitude on this part of Mr. Gillman's Dissertation, and would impress our readers with the propriety of rejecting an opinion founded on loose and popular tradition rather than on an observance of nature. Without the verification of the disease being propagated by the *saliva* of the dog, in the case related by Mr. Gillman, it would,

* Vide Note, page 240.

perhaps, perhaps, be denied that he was destroyed by *rabies.* It is plain, therefore, that we have yet to learn the generic marks of this disease.

There are other facts connected with this detail, of no small importance, and which would have been lost to science but for the vigilance of our author. The varying periods at which the disease appears after the inoculation, is distinctly marked in these instances. In one pig the disease appeared on the 10th day after the bite; in the other on the 14th; and the sow was attacked on the 27th day. In two of these cases there was evident increasing irritation in the bitten part: it was seen in the pig to precede the symptoms twenty-four hours, and also in the sow, probably, though unobserved until the general affection had appeared.*

It is known with great certainty, that the specific poison of rabies exists in the *saliva;* but it has been a question how far the solids and fluids have been generally contaminated. This history furnishes some facts which go far to prove that the infecting material of rabies is hardly to be found but in the saliva; and certainly a morbid fluid is no more to be detected in the other fluids of the body, than is the natural venom of the viper or *crotalus horridus.*

"As I wished (says Mr. Gillman) to prove, if possible, whether the fluid contained in these apparent pustules (on the villous coat of the stomach of the dog whose case has just been related) had the power of infecting other animals, I inoculated two rabbits with it in

---

* The time at which the disease appears in various individuals after the infliction of the bite, is known to be indeterminate. It is desirable to ascertain the shortest and the longest period at which rabies has occurred after the insertion of the poison. We insert the following history to assist in determining this question. On the 8th of June, 1791, the man who slept in the kennel, and had the care, of Earl Fitzwilliam's hounds, was in the night unusually disturbed by the hounds fighting. He got up several times to quiet them, but always found the same hound quarreling. He was induced in consequence to notice him; and, finding him stupid and quarrelsome, he confined him by himself: the hounds were quiet for the remainder of the night. At the end of the third day he became rabid, and on the fifth died. Preparations were made for confining the forty-two couple of hounds separately. The symptoms and progress of the disease were exactly minuted by a medical gentleman. Six of the hounds became rabid in the following order:—the first on July the 1st; the second on August the 3d; the third on September the 3d; the fourth on September the 4th; the fifth on November the 10th; the sixth on December the 8th.—*Daniel's Rural Sports.*

several several places, but without producing the disease or affecting the health of the animal apparently in the slightest degree."

The sow and two pigs which were bitten by this dog, and died rabid, were dissected by Mr. Brooks the anatomist; and we observed, that, when employed in this process, his fingers had several slight wounds upon them: but no inconvenience occurred. The flesh of these rabid animals was also perfectly harmless: it was eaten by dogs, foxes, eagles, and hawks, with impunity. A rabid dog was examined after death by Mr. Bayford, of Parson's Green. While inspecting the fauces of this dog, Mr. Bayford cut his finger, but without subsequent disease. The apprehension, therefore, that considerable danger is incurred by anatomists in the dissection of rabid animals, seems unfounded.

The remaining part of this section treats of the remote or primary cause of rabies, and of its spontaneous origin: but, as the author's reasoning turns upon facts selected from printed documents, already in the hands of medical readers, we shall pass it over with observing that the occasional causes from which this disease is said to arise are climate—putrid aliment—deficiency of water—want of perspiration—and worm under the tongue; and shall conclude, thus far, with citing the subsequent corollaries.

"1st. That wildness, fury, madness, &c. which the term *rabid* implies, does not form an essential character of the disease.

"2d. That the dread of fluids, in consequence of which by some writers this disease has been termed *hydrophobia*, is not an essential symptom, nor is the loss of appetite; but, on the contrary, dogs eagerly lap fluids, although in some period of the disease they are deprived of the power of swallowing them; and they will frequently as freely eat.

"3d. That appearances of inflammation, particularly of the stomach, are not always found after death; and that the bodies of these animals occasionally exhibit no mark of disease whatever.

"4th. That, although the preceding symptoms may be absent, as fierceness, loss of appetite, and dread of water, and though there should be no mark of disease after death, &c. the dog is capable of communicating this disease to various animals, particularly to the human species.

"5th. That climate, putrid aliment, want of water, deficiency of perspiration, &c. are from the best authorities not the cause of rabies.

"6th. That there is not evidence sufficient to disprove that this disease arises spontaneously in dogs, but that neglect of cleanliness and confinement may be considered as highly contributing to the production of this dreadful malady.

"7th. That the proposals for quarantine, but for a much longer period, period, are deserving of consideration, and may tend at least to develop some important points.

"8th. That all persons should avoid familiarity with strange dogs, and never trust or fondle any dog when he has deviated from his general appearances or habits, or is out of health.

"9th. When a person is bitten, the dog should be confined for ten days, and not killed immediately, as is too often practised, in order that a correct opinion may be formed of the case."

In the second section, "*On the Treatment of the Bite of a Rabid Animal,*" the author goes into a question of the deepest importance. In rabies, when once manifested in the system, all human skill, all power of medicine, has failed: to prevent the accession of, rather than study for a remedy for, the disease now actually existing, should then be the point to which all our views ought to be directed. It is matter of curious speculation, rather than of practical utility; proper to agitate the reasoning pathologist, more than to influence the plain therapeutist, whether the rabid "virus is mixed with the blood through the medium of the lymphatics, which absorb the poisonous saliva, and thus in a secondary manner, acting on the nerves, producing this fatal disorder: or that the infection acts locally, and by irritation, not only on the nerves, but tendons also, sympathetically affects the whole nervous system, without the introduction of the poison into the circulation." Whichever of these be the fact, the effect is the same; always resisting medicine, always terminating in death. To prevent this absorption, or this irritation without absorption, by removing the deleterious body, is, therefore, the great and only object.

To effect this, various means have been suggested. Scarification, suction, caustic, ablution, and excision, are those which most deserve attention; and of these our author inquires into the *modus operandi* and comparative efficacy of three—caustic, ablution, and excision.

To the employment of caustic he objects both theoretically and practically. The frequent accession of rabies after the application of these substances to the wound, is doubtless a forcible argument against trusting to them; and many cases are cited to establish this fact. But whether the reasoning intended to explain this result be satisfactory, we shall submit to our readers; doing the author, however, the justice of allowing him to state his own conclusions. After a short inquiry into the chemical composition of the saliva, and the structure of the muscular fibre, the affinities to which they are liable, and the decomposition they are subjected to, he observes,

"Whenever alkalies, as pure potash, or pure potash and lime, are inserted inserted into a wound containing the poisonous saliva, and there, as is generally practised, rubbed about for some little time, the alkali first unites with the morbid saliva, next with the more muscular or solid parts, &c. of the wound, till the whole of the surrounding parts, as far as its action extends, are intimately blended. A new compound is in consequence formed, a saponaceous mass, or eschar, which is generally supposed to remain until it sloughs away. Of what then is this new compound formed, but of dead animal matter, a caustic, and the peculiar poison which we believe to be the cause of hydrophobia *(rabies)*, and which it ought to be our immediate care to remove? It is true the neighboring absorbents are destroyed as far as the action of the caustic extends; but the canine virus is as likely to extend with it, being only in a state of union from the commencement of this operation, which, if continued, the poison is uniformly dispersed through the whole of the adjacent parts, forming an animal soap by their commixture. Hence, by such means, a more extended surface is exposed to the action of the absorbents, which are rendered highly irritable, and more active, and in consequence, perhaps, the case becomes more desperate."

"The same argument will equally hold good with regard to the other powerful solvents, as the acids, and the acid preparations, if the poison be not decomposed or removed. If they should destroy the absorbents about the wound, they, previously to this, dissolve the morbid saliva; therefore, the sound parts are likely to become contaminated by them the moment they enter the wound, and hold the virus in solution; for by corroding the muscular parts the poison is diffused. If perchance this poison should happily be discovered to be some peculiar saline compound, or if the poison should be rendered inert, when mixed with the agents already referred to, we might then hope every thing from the application of either acid or alkaline preparations, as we certainly know they possess the power of destroying most organic compounds: but, as we are still utterly ignorant of the chemical nature of this poison, let us prefer imitating and following those methods which appear to present the more wished-for and desired success."

We come now to notice "those methods which appear to *present* the desired success." These are EXTIRPATION and ABLUTION. It is self-evident that the entire removal of the contagious material will afford the most perfect security, and this is effected with greater certainty by excision of the bitten part, than by any other process. But excision is an operation, when it can be employed, of great nicety and care; not the mere cutting away the part with clumsy haste.

"Much caution and judgment is requisite in excising the bitten part. I have known of failures (and there are some recorded by Dr. Hamilton) where the parts were excised, and afterwards cauterized; but I am fully persuaded that this arises from want of sufficient attention to some of the minuter circumstances of the operation.

"The first thing requisite, before the excision of the bitten part, is to wash not only the inside of the wound, but also the surrounding parts, with great care; for, if this be neglected, and the poisonous saliva be not removed, in making incisions on each side of the wound, the sound parts through which they are made will be inoculated with the virus. Two incisions should then be made, one on each side of the wound, forming an elipsis in such as will admit of it, which should be carried to such a depth as completely to remove the part. It should then be carefully examined if there is any part in the piece excised through which the dog's tooth appears to have passed; and in case there is, the excision should be carried deeper. In making the incisions, great attention should be paid to the direction of the tooth; and, if the knife should enter the wound made by the dog's tooth, I should consider it always necessary to recommence the operation with a clean knife, and this as often as the occurrence should take place: for, if we continue to use the same knife, which is likely to be contaminated in consequence of its entering the wounded parts, the operation may be rendered useless by the sound parts becoming inoculated with the canine virus."

These ample directions for the excision of the bitten part, are sufficiently impressive and judicious, we trust, to be indelibly fixed on the operator's mind. To little purpose, indeed, will the surgeon expose his patient to fear and pain, if with his knife he transfers the poison to the parts through which his incision passes. We are so fully convinced of the possibility, and even the probability, of this, that we feel indebted to our author for putting the case with such precision and force. We shall make no apology for pursuing this important part of the prophylactic treatment a little further. Mr. Gillman has given very proper directions for the operation of excision, and has increased the value of these directions by cautions which have left his statement scarcely susceptible of improvement. But there is still a serious question connected with this process: At what period after the infliction of the wound should excision be employed? *Immediately.* Various impediments, however, arise to prevent this. At what period then after the bite can it be used with a prospect of success? there is great reason to believe at any time after, even when symptoms of rabies have arisen.

"In the determination of the question (says Mr. Gillman) as to the time when extirpation of the parts infected by the bite may be performed, it is of great importance to consider whether the poison does always remain in the substance where it was first infused, until by inflammation, or by some other cause, a sufficient dose is generated to infect the whole system. Indeed, the notorious connection between a painful or inflamed state of the original wound immediately preceding the constitutional symptoms, warrants the supposition, and points out the expediency of removing or destroying the parts to the last."

So

So far as our own observation and experience have gone, they have met this conclusion. Not more than two years since a man came under our care, who was bitten by a dog believed to be rabid. A few hours after he received a wound on his hand, the bite was cut out, and certainly without the precautions suggested by our author. The wound soon healed. In six weeks from the bite he again applied to us. The cicatrix was now elevated, had changed to a livid hue, and he felt a tingling sensation in the part where the wound had been. Ample extirpation of the part, with an inch and a half of the surrounding sound substance, was had recourse to. He has since felt no inconvenience; neither has the second cicatrix ever assumed the livid hue of the first. It cannot be said, positively, that this person would have fallen into rabies; the symptoms were too threatening to trust to the chance; and the fair conclusion is, that he was saved by the second excision.

It sometimes happens that the bite is so placed as not to admit of excision. In this dilemma the resource is, if we reject caustic, a careful and persevering ABLUTION. The rationale of this is obvious; but there may be some question as to the fluid to be employed. When the choice is not influenced by circumstances, our author uses a weak solution of volatile alkali, in the proportion of one part of the alkali to four of water. With this solution,* (fully capable of dissolving the saliva), the wounded parts should be freely washed, and injections, with a syringe, forcibly made into the wound. After this has been persevered in for a considerable time, it is proposed to use warm water to promote a flow of blood, which may assist in washing away any remaining particle of the poison.

The method here laid down cannot be objected to, but it seems to admit of being extended upon the same principle, both by a combination and arrangement of the several means proposed, and by the introduction of an auxiliary of which Mr. Gillman has noticed the name only; we mean the exhausted receiver or *suction*. On this last we have one remark to make. If the hazard, incurred by an application of the lips to the wound, be a valid objection, and perhaps it is, it does not extend to the use of cupping-glasses? If after

* Mr. Gillman ascertained this proportion to be fittest for the purposes of ablution: when stronger it is observed to corrode the solid parts, and probably hold the virus in solution. It is therefore rejected on the principle that induced him to reject caustic, as previously stated. While employing ablution, he suggests the propriety of using clean sponges.

excision, or before, or at any period, it be an object to promote a full flow of blood from the wound, how can this be effected more certainly than by suction? We have therefore no hesitation in recommending cupping, as one, and possibly not the least, effectual part of the prophylactic process.

From a view of the subject then, as stated by our author, and from our own reflections and observations upon it, we shall venture to suggest the following routine, as affording a fair prospect of security from the dreadful effects of this poison. Immediately on the infliction of the bite, assiduous ablution with the first water that can be found, either with or without soap, and this to be continued unceasingly until professional aid is procured. On the arrival of the surgeon, full excision of the bitten part, according to the directions before stated, if the circumstance of situation, or other objections, do not forbid. After excision, ablution again with solution of volatile alkali in water; and, when the flow of blood begins to cease, suction with the cupping-glass. The alternate employment of ablution and the exhausted receiver to be continued many hours. We forbear to fix on a definite time, that must be left to the discretion of the surgeon employed; but we do not know why it should not be extended to twenty-four hours. The object is of the last importance; time and trouble are minor considerations. After proceeding thus far, why should not caustic, or some irritating material, be applied to the wound, so as to produce a slough, in the first instance, and afterward a purulent discharge, for some weeks? Both analogy and very fairly-stated facts countenance this part of the process. It is well known that inoculation for the small-pox succeeds with most certainty when the puncture is slight, and when no inflammation or morbid action ensues until excited by the virus. On the contrary, when much disease takes place in the part where the variolous fluid is inserted, from any other cause than the stimulus of that fluid itself, it is also well known that the chance for infecting the system, suffers a reduction agreeing with the degree of adventitious inflammation. In the case of Bellamy (Med. Observ. and Inquiries,) the wound was small, and soon healed,—he died rabid. In the case of his servant, bitten by the same animal and nearly at the same time, the wound ulcerated and could not be healed for many weeks,—she recovered without any symptoms of rabies. We do not state this as a conclusive fact; but it is strong presumptive evidence, supported by close analogies.

The objections to the use of caustics, drawn from or supported by the chemistry of the day, though ingeniously put,

3 carry carry with them no conviction. The chemistry of life, if the expression may be permitted, differs so much from that of the crucible and the furnace, that facts drawn from the latter, with a view to explain the principles and actions of the former, lead into endless labyrinths of fallacy and error. We agree thus far with our author, however, on the subject of caustic: it should never be a primary application, but, for reasons previously stated, should close the prophylactic process.

Upon the preventive remedies, supposed to act on the system, we must not waste time. From the *Pulvis Antilissus* of Dr. Mead, to the clumsiest *Mad-dog drink* that cunning ever imposed upon credulity, all is mistake or deception. They have always been worse than useless—they have sunk the patient into idiotic security—and the dreadful symptoms of rabies have awakened him, too late, to a sense of his condition.

The third and last section treats of the "*Consequences of the Bite of a Rabid Animal.*" Over this we shall rapidly pass. It is painful to record events unsus[illegible]ined by a single instance of success. Every remedy employed against the disease, excited by the rabid virus, has hitherto proved inert, even to moderate the symptoms. To recount their names, doses, and effects, is but giving a musterroll of deaths. In consonance, however, to the principle which has influenced us throughout our observations on this Dissertation—that of presenting to our readers those facts which the author has taken from nature, we shall cite a case of rabies which fell partly under his own observation, and is partly copied from the notes of Mr. Scudamore.

"December 13th, 1807. James Anderson, aged 14, of slight unhealthy aspect, subject to frequent head-achs, was yesterday at noon attacked with rigors succeeded by hot fits, and this morning (Sunday) had all the symptoms of hydrophobia, of which he died the night following. About an hour before his death, he had an interval of reason, and gave a full account of the accident. From him and from his mother were learnt the following particulars of the case preceding the attack:—A dog ran into the stable which he was cleaning, and bit him in the under lip. A few days after he complained to his mother that it pained him. She examined it. Being ignorant of the cause, and considering it only a chop, she applied tallow to it, and no further notice was taken of the wound. In about a week or ten days he complained of giddiness and pain in his head, which, as he was subject to, gave no alarm. About this period also he began to spit unusually: this continued for a fortnight, so as to excite his mother's fears lest he should be consumptive. He passed his nights indifferently, and in uneasy sleep. On Wednesday, three weeks subsequent to the bite, he made serious complaint complaint of his head and the wounded part, which was on the left side: it throbbed with violence, the pain extending to the ear. The mother, considering these symptoms as only a cold, applied camomile fomentations to the side of the face, and gave him some warm gin and water, which appeared to relieve him. He then went to bed, but slept little, and continued occasionally to complain of his head. At this time, however, and during Thursday and Friday, he ate and drank without any inconvenience. On Saturday he lost his appetite; and, while employed in cleaning his horse, which it was his occupation to drive about in a water cart, he felt alternately cold and hot, and was obliged to discontinue his employment. He complained of thirst; the phlegm was viscid, which he spat vehemently, desiring those around him to remove, as he feared they would suffocate him. During the night he made a violent attempt to get out of bed, which those around him resisted. He very angrily remonstrated with them, but soon became calm. This morning (Sunday) his mother, being alarmed, sent to Mr. Scudamore, who found that he had passed a restless night, was extremely agitated, had great anxiety of breathing, and complained of a slight sense of constriction across the upper part of his chest. His eyes were suffused, and the pupils dilated; tongue dry, and furred in the middle, but moist at the edges; pulse from 80 to 100, quickly varying; countenance melancholy, and expressive of great distress; had much of the characteristic spasm in attempting to swallow. Calomel and opium were administered. Three P. M. Mr. Scudamore saw him again; pulse 90; he had taken eight grains of calomel and six of opium, and had swallowed also about half a pint of toast and water, but with great difficulty and distress, requiring his head to be held. Six P.M. I first saw him. He was dozing; but, on my entering the room, he was instantly seized with a convulsive sigh, and sprung up in the bed suddenly. He was perfectly rational, but spoke in a melancholy tone, and complained much of sickness, which he said prevented his drinking. He had a slight pain about the scrobiculus cordis, but no pain whatever about the throat. When requested to drink, he was again seized with a convulsive inspiration, and answered quickly he would do it if he could. He then called to his mother to hold his head, seized a cup which was offered to him filled with toast and water, and with many convulsive sighs, and with much difficulty, swallowed the greatest part of the fluid contained in it. He complained soon again of thirst, but said the sickness at his stomach prevented him from drinking any more. Half a lemon lying on a table in the room, I requested him to squeeze a little of the juice into his mouth; but he complained again of much sickness, and would not attempt it, though his thirst was great. However, as I wished him to rub his tongue only with the lemon, he at length consented; but he had as much difficulty in carrying it to his mouth, and placing it on his tongue, and was nearly as much agitated, as in swallowing the toast and water. He appeared drowsy from the opium, and was disposed to sleep; but, when he lay down for a few minutes and attempted to doze, doze, he was suddenly seized with a convulsive inspiration, and as suddenly jumped up in the bed, called for his mother, and was much agitated and distressed. At eight o'clock I saw him again. He said his head was much relieved since the morning; suffered no one to touch him but his mother. Pulse 80, extremely tremulous. Eleven at night I repeated the visit. He had passed a pint of urine, was still sensible, but much agitated, and sighed more frequently. Pulse 90, hard and tremulous. Any new question that was now put to him, the least motion in the room, the drawing the bed curtains, stirring the fire, or any little noise whatever, was immediately succeeded by a convulsive sigh, and increased his anxiety and distress. The proposition of placing him in the warm bath so alarmed him, that it was not put into execution. The nausea still continued; he could not be prevailed upon to drink, and was much distressed with flatulence. Twelve at night, all the symptoms the same, and he knew those around him. Mr. Scudamore again proposed to place him in the bath. He assented to the proposition; heard, as he observed, the reasoning upon it mildly, but requested him in the most anxious manner to wait. When he offered him drink, he refused it quickly and emphatically, but in terms of great civility. He passed his urine a little at a time. After considerable persuasion, he got from the bed, but would not go into the bath: had no intestinal evacuation. Took his powders nearly every hour, which consisted of three grains of calomel and three of opium. Mr. Scudamore's assistant remained with him during the night, who reported, that he was more tranquil till half past two, when six grains of calomel and opium were given him in jelly, which he had great difficulty in swallowing. Dozed, but was disturbed with frequent spasms, till near four. He then became extremely restless. Pulse 120. When toast and water was offered him, it increased his agitation, and his pulse to 132. Asked at five o'clock for toast and water, which increased his spasms violently. Pulse 100.

"Monday, December 16th. All symptoms the same as yesterday; spasms as frequent, but more feeble; continued the opium and calomel as often as he could be made to swallow. Musk and camphor were now administered by the advice of Drs. Marcet and Yelloly, who visited him, but he could not be made to swallow more than one dose. In the evening he was much exhausted by the disease: he called frequently for tea, which he drank eagerly, but with much agitation. He succeeded in swallowing several small tea-cups full, and ate a small quantity of cake. He seemed pleased at the efforts he had made in swallowing, exclaiming "well done! at it again;' but vomited all he took soon after. He now became so extremely irritable that he would not allow his mother, nor in short any thing, to touch him. These symptoms increased till within two hours of his death, when he had a calm interval, and requested to see his companion who was bitten at the same time with himself. He then related the circumstance of his 'having been bitten by a large dog, as big as the coachman's, with long ears that reached to his nose.' At length he gradually sunk; his hands and and feet became cold; he rose up in his bed, fell back, muttered, and expired."

We could add to the value of this article by inserting, from the appendix, a well-written case of *Rabies*, by Mr. Carlisle; but we have already, perhaps, notwithstanding the interesting nature of the subject, trespassed too much on the time of our readers, and must hasten to a conclusion.

The impression made on our minds, upon reading this Dissertation, was, that of its being the production of a man of strong intellect, and a close observer of nature. The facts he relates from his own knowledge are interesting generally, and sometimes have an air of novelty. The history of the appearances in the stomach of the rabid dog, accompanied with a plate, we believe to be new, so far as regards any printed account illustrated by an engraving. His cases are detailed with minute precision, and he has added to the prophylactic process important precautionary directions. His selections from other writers, made to fill up the picture, will be useful to the tyro; but those who have left the schools will only be impressed with the result of his own individual knowledge, as having the chance of communicating new facts, and presenting novel illustration. With his style we are not quite satisfied, and we could object to some of his conclusions. The cure of rabies he has left as he found it—hopeless; but he has enforced, most judiciously, the prophylaxis. We must consider this Dissertation to be a valuable addition to the stock of medical knowledge on Rabies Canina.

## Figures and Tables

**Figure f1:**